# One Hundred Consecutive Cases of Skin Disease, V

**Published:** 1891-05

**Authors:** M. B. Hutchins

**Affiliations:** Atlanta, Ga.; Lecturer on Diseases of the Skin, Atlanta Medical College


### ONE HUNDRED CONSECUTIVE CASES OF SKIN DISEASES.

### V.

BY M. B. HUTCHINS, M. D.,
Lecturer on Diseases of the Skin, Atlanta Medical College.

[NOTE.—In preceding article there are a few typographical errors, the worst being in the last prescription, where "Lanolin *pulv.*" should, of course, read, "Lanolin *pur.*" (pure), which I overlooked in the proof.]

### ACNE VULGARIS.

The time of occurrence of this form of acne is put between the ages of fifteen and thirty, the disease usually beginning at puberty. Beginning on the face, as a rule, about this time, it may subside or disappear by twenty-five, or may then have begun to appear on shoulders, back and chest. The youngest patient with this disease, which I recorded, was nineteen, the trouble having been present since fourteen. The oldest was a man of twenty-eight, who had an occasional lesion on the face, but a most severe outbreak on the back and shoulders. The disease began on his face at sixteen, on chest at eighteen, an occasional lesion still occurring there ; and on back it appeared at twenty-two. After six months' most faithful following of the treatment he was entirely free of the disease. A particular type of acne lesion did not occur in the majority of the cases, but there was present every form of papule, pustule or nodule which occurs in acne, from the simple "black head," the small papule or papulo-pustule, acutely or indolently inflammatory, to the large finger nail, or larger-sized, firm, inflammatory mass, containing only sebaceous matter, or, later, pus. The disease belongs essentially to the indolent inflammatory processes, and I have not seen a case which I thought required preliminary, "sedative" treatment.

Here, as in the description of seborrhoic eczema, I confess to using scarcely more than one remedy, of course varying the strength as the skin required. Below is the "average" :

R. Zinci sulphatis,

Potasii sulphidi,

Sulphur. precipitat., aa. ʒi.

Aquæ, ℥iv.

M. Sig.—Shake, apply and let dry.

This must be made with regard to chemical reactions. Sometimes half the fluid may be alcohol—in order, for one thing, that the application may dry more quickly. The "rotten egg" odor may be partially suppressed by the use of rose, instead of plain, water.

Constipation was practically the only deviation from normal health, nothing else, as a rule, being found "wrong." Constipation was treated with the "cascara and nux" mixture already described, and generally this treatment was successful. Where slight "dyspepsia" was noted simple "vegetable tonics" were prescribed, but the majority of cases neither received nor required internal treatment.

Besides the application of the lotion above mentioned, I have found pressing out the "black heads" (comedones), puncturing every lesion without regard to its stage, form or character, and pressing out contents—whether simple sebum or pus—to be extremely useful in hastening the decline of the eruption. Applications, at night, of very hot water to affected skin had a pleasant and useful tonic effect. If much greasiness and oiliness of the skin were present, an aqueous alcoholic lotion, containing three per cent. of resorcin, was found useful. On that severe case of the back, frictions, with

R. Saponis viridis, ℥ii,

Sp. vin. rect., ℥i,

M., were found beneficial as a local stimulant and to remove effete epidermis.

The disease is a kind of physical barometer, tending, as it does, to relapse after the occurrence of a slight indigestion, constipation, or such variation from health.

Various dermatologists, quoting the view that sexual continence had some influence in the production of the disease, I made inquiry concerning the personal habits of the majority of the (male) patients, and found continence rather lightly practiced. It is more likely that irregularity in the sexual life has an influence. Masturbation was not inquired into.

Persistence in treatment was rewarded by recovery ; the patients who treated themselves spasmodically are to be assured that they will *outgrow* the disease some day.

ROSACEA—ACNE ROSACEA.

Of these cases I find three recorded. It is difficult to differentiate between the two affections, for we find slight evidence of the acne in the majority of the rosacea cases, thus rendering the designation *acne rosacea* appropriate. This disease is commonly found to begin about the age when common acne should disappear, but I had one case, the driver of a delivery wagon, age twenty-one, in which simple acne, chiefly composed of comedones and small papules, was present, and, besides, a condition of dilatation of the capillaries of the skin—probably due to exposure to all sorts of weather. Quite a typical case of acne rosacea was that of a lady of thirty-two. There was flushing of skin of cheeks and nose, end of nose a dull red, and dilated vessels, with here and there a pin-head sized, indolent papulo-vesicle, or pustule, simulating minute acne lesions. Follicles of nose were dilated, and many were filled with dried sebaceous matter. Skin of face was oily, and there was considerable dandruff on the scalp. Uterine derangements existed, sufficient to produce sterility.

The seborrhoic condition was treated with the *lotio resorcin;* the acne rosacea directly, with the lotion for acne vulgaris, omitting the sulphur. Vessels which are too much dilated to yield to treatment with lotions, etc., can be destroyed by electrolysis, just as the dilated vessels in "birth marks" can be destroyed. In acne rosacea it is important to find out if there be functional derangement of any kind, to direct internal medication towards its removal. The hot water applications, as in common acne, are also every useful in this disease. Protection from sun, wind and artificial heat are essential. The cases require long continued and persistent treatment, and deviations from health must be remedied. Finally, the possession of a red face or red nose is more frequently due to other causes than to indulgence in alcoholics.

16½ *Whitehall St.*

---

Lawson Tait says: Where syphilis kills its tens, gonorrhœa kills its thousands; and it would take the sufferings of a hundred cases of syphilis to make up for the long, weary years of agony of one case of gonorrhœa pyosalpinx.—*N. Y. Medical Times.*

---

The State Medical Society of Michigan has had a bill introduced into the legislature to regulate the practice of medicine in that State. It seems to have been carefully drawn, and deserves to become a law.—*Medical Record.*

